# Predicting unplanned readmission due to cardiovascular disease in hospitalized patients with cancer: a machine learning approach

**DOI:** 10.1038/s41598-023-40552-4

**Published:** 2023-08-18

**Authors:** Sola Han, Ted J. Sohn, Boon Peng Ng, Chanhyun Park

**Affiliations:** 1 Health Outcomes Division, College of Pharmacy, The University of Texas at Austin, Austin, TX 78712 USA; 2 College of Nursing, University of Central Florida, Orlando, FL USA; 3 Disability, Aging, and Technology Cluster, University of Central Florida, Orlando, FL USA

**Keywords:** Risk factors, Health services, Public health, Cancer, Cardiovascular diseases

## Abstract

Cardiovascular disease (CVD) in cancer patients can affect the risk of unplanned readmissions, which have been reported to be costly and associated with worse mortality and prognosis. We aimed to demonstrate the feasibility of using machine learning techniques in predicting the risk of unplanned 180-day readmission attributable to CVD among hospitalized cancer patients using the 2017–2018 Nationwide Readmissions Database. We included hospitalized cancer patients, and the outcome was unplanned hospital readmission due to any CVD within 180 days after discharge. CVD included atrial fibrillation, coronary artery disease, heart failure, stroke, peripheral artery disease, cardiomegaly, and cardiomyopathy. Decision tree (DT), random forest, extreme gradient boost (XGBoost), and AdaBoost were implemented. Accuracy, precision, recall, F2 score, and receiver operating characteristic curve (AUC) were used to assess the model’s performance. Among 358,629 hospitalized patients with cancer, 5.86% (n = 21,021) experienced unplanned readmission due to any CVD. The three ensemble algorithms outperformed the DT, with the XGBoost displaying the best performance. We found length of stay, age, and cancer surgery were important predictors of CVD-related unplanned hospitalization in cancer patients. Machine learning models can predict the risk of unplanned readmission due to CVD among hospitalized cancer patients.

## Introduction

Cardio-oncology is an emerging field for improving screening, prevention, and management of cardiovascular diseases (CVD) that are caused by various pathophysiological mechanisms in cancer patients^1^. Growing evidence suggests that cancer and CVD have various overlapping risk factors or underlying mechanisms^2–4^, and cancer treatments can be associated with developing a wide spectrum of CVD (e.g., arrhythmia, coronary artery disease, heart failure, peripheral artery disease, or stroke)^4–6^.

In terms of quality of care, unplanned readmission has become a well-recognized objective metric in healthcare. CVD in cancer patients can affect the risk of unplanned readmissions, which have been reported to be costly and associated with worse mortality and prognosis in cancer patients^7,8^. To facilitate value-driven healthcare decision making to implement cardioprotective strategies or to adjust interventions, there is a need to identify cancer patients who are at a higher risk of unplanned CVD readmission. However, there is currently no risk prediction model for unplanned CVD readmission in cancer patients.

In earlier studies, machine learning (ML) approaches have been shown to improve the prediction of CVD risk and incidence over traditional CVD risk scores (e.g., scores from the American College of Cardiology or the American Heart Association), which may be driven by the flexibility of ML models^9,10^. Additionally, a variety of ML algorithms have been used in predicting CVD and CVD-related hospitalizations^10,11^. Therefore, we hypothesized that ML methods could likely provide reliable predictive information about the future risk of unplanned CVD readmissions in cancer patients.

Therefore, using hospital discharge data, we aimed to demonstrate the feasibility of using ML techniques in predicting the risk of unplanned readmission due to CVD among hospitalized cancer patients. In addition, the secondary objective is to compare the predictive performance of different ML algorithms and identify the important risk factors contributing more to the model prediction performance. Based on the previous study reporting the period of increased CVD risk in cancer patients^12,13^, we used a risk of 180-day readmission for CVD to fully capture the elevated risk of CVD among cancer patients.

## Methods

### Data source and study population

We retrieved the information of hospitalized patients with a primary diagnosis of cancer (Clinical Classifications Software Refined categories of NEO001-NEO071) from the 2017 and 2018 Nationwide Readmissions Database (NRD). The NRD is designed to generate nationally representative estimates of hospital readmissions and is part of the Healthcare Cost and Utilization Project (HCUP) that is sponsored by the Agency for Healthcare Research and Quality^14^. All methods were carried out in accordance with relevant guidelines and regulations. Because HCUP-NRD is publicly available deidentified data, the Institutional Review Board of The University of Texas at Austin exempted the study and informed consent was not required.

Patients younger than 18 years of age, patients with any listed diagnosis of CVD (atrial fibrillation, coronary artery disease, heart failure, stroke, peripheral artery disease, cardiomegaly, and cardiomyopathy) or who died in the index hospitalization for cancer, patients with missing values in features, or patients discharged between July and December (as these hospitalizations would lack a minimum 180-day follow-up data) were excluded. Supplementary Fig. 1 depicts the sample selection flow.

### Outcomes

Our primary outcome was an unplanned CVD readmission event within 180 days after the index hospitalization for cancer. Unplanned readmissions were identified by excluding elective readmissions using the elective variable in the database. International Classification of Disease, Tenth Revision, Clinical Modification (ICD-10-CM) codes were used to define CVD, including atrial fibrillation (I48), coronary artery disease (I20–I25, I252), cardiomegaly (I517), cardiomyopathy (I43, I427, I429, I420, I425), heart failure (I50), peripheral artery disease (I70, I74, I739), and stroke (I60–I63, I65–I66, I69, I672, I679, I6781–I6782)^15^.

### Features (predicting variables)

We included features that are commonly applied in related studies to predict risk of readmission: age; sex; median household income level; primary payer; All Patient Refined DRG (APR-DRG): risk of mortality subclass; APR-DRG: severity of illness subclass; weekend index admission; Elixhauser index for the risk of readmission^16^; length of stay and cost of index admission; hospital bed size; smoking; hyperlipidemia; previous percutaneous coronary intervention (PCI); previous coronary artery bypass graft (CABG); previous transient ischemic attack (TIA) or stroke; thrombocytopenia; chronic renal failure; anemia; coagulopathies; liver disease; long term (current) use of anticoagulation; long term (current) use of antithrombotics or antiplatelets; cancer surgery; radiation; chemotherapy; metastasis; and cancer types (i.e., colorectal, lung/bronchus, melanoma, breast, uterus, prostate, bladder, kidney/ureter/renal pelvis, non-Hodgkin lymphoma, leukemia, and other).

### Machine learning algorithms

To build the prediction models, we used four commonly used tree-based ML algorithms: decision tree, AdaBoost, extreme gradient boosting (XGBoost), and random forest. We chose these four algorithms because these have been applied commonly and performed well in the CVD research field^10^. Decision tree is a classifier that is constructed by repeatedly splitting the training set using features to predict the class labels^17^. AdaBoost is an ensemble classifier that trains weak classifiers and improves the performance of these classifiers by increasing the weight of misclassified samples as the steps progress^17–19^. The XGBoost is an ensemble classifier that successively trains weak classifiers to correct the errors of the last prediction until there is no further improvement^17,20^. Random forest is an ensemble classifier that trains multiple weak classifiers by repeating random sampling for predictors and observations; the final outcome is determined by a majority vote method^18,20,21^. The models were built using Python (version 3.9.12; Python Software Foundation) and several Python modules (numpy, pandas, matplotlib, sklearn, imblearn, and xgboost).

As shown in Table 1, our dataset was highly imbalanced, with only 21,021 (5.86%) CVD readmissions after the index admission for cancer. We used the cost-sensitive learning approach to deal with imbalanced data in our study^22,23^. For the analyses, we considered unplanned readmissions related to a composite of CVD, which included atrial fibrillation (6623 cases), coronary artery disease (7313 cases), cardiomegaly (367 cases), cardiomyopathy (1169 cases), heart failure (5782 cases), peripheral artery disease (2034 cases), and stroke (4508 cases). The hyperparameter search spaces for each model and the best hyperparameters resulting in the highest performance on the test set are shown in Table 2. The feature selection method using L1 penalty is implemented to identify the best set of features.

**Table 1:** Baseline features of the study cohort.

	Cancer (N = 358,629)
	N (%)
Age, mean (SD)	62.20 (13.39)
Age, median (IQR)	63 (54–71)
Sex	
Male	173,972 (48.51)
Female	184,657 (51.49)
Income	
0–25th	85,391 (23.81)
26–50th	92,655 (25.84)
51–75th	91,062 (25.39)
76–100th	89,521 (24.96)
Insurance	
Medicare	161,725 (45.10)
Medicaid	43,066 (12.01)
Private insurance	136,803 (38.15)
Other	17,035 (4.75)
APR-DRG: severity of illness	
Minor loss of function	77,696 (21.66)
Moderate loss of function	142,334 (39.69)
Major loss of junction	109,583 (30.56)
Extreme loss of function	29,016 (8.09)
APR-DRG: risk of mortality	
Minor likelihood of dying	145,117 (40.46)
Moderate likelihood of dying	118,137 (32.94)
Major likelihood of dying	79,100 (22.06)
Extreme likelihood of dying	16,275 (4.54)
Weekend index admission	35,650 (9.94)
Elixhauser index, mean (SD)	4.98 (5.67)
Length of stay (days) of index hospitalization, mean (SD)	4.14 (5.24)
Cost of index hospitalization, mean (SD)	21920.53 (25096.83)
Hospital bed size	
Small	40,110 (11.18)
Medium	80,419 (22.42)
Large	238,100 (66.39)
Clinical conditions	
Smoking	52,161 (14.54)
Hyperlipidemia	98,369 (27.43)
Previous PCI	499 (0.14)
Previous CABG	920 (0.26)
Previous TIA/stroke	9749 (2.72)
Thrombocytopenia	16,084 (4.48)
Chronic renal failure	25,130 (7.01)
Anemia	81,330 (22.68)
Coagulopathies	21,725 (6.06)
Liver disease	18,033 (5.03)
Anticoagulation	9965 (2.78)
Antithrombotics/antiplatelets	1621 (0.45)
Cancer surgery	118,312 (32.99)
Radiation	5831 (1.63)
Chemotherapy	14,793 (4.12)
Metastasis	104,047 (29.01)
Cancer types	
Colorectal	46,568 (12.99)
Lung/bronchus	33,527 (9.35)
Melanoma	701 (0.20)
Breast	20,923 (5.83)
Uterus	1711 (0.48)
Prostate	31,224 (8.71)
Bladder	7884 (2.20)
Kidney/ureter/renal pelvis	19,517 (5.44)
Non-hodgkin lymphoma	11,240 (3.13)
Leukemia	10,042 (2.80)
Other	175,292 (48.88)
Outcome: unplanned CVD readmission	
Yes	21,021 (5.86)
No	337,608 (94.14)

**Table 2:** Range of the evaluated hyperparameters for the machine learning models.

Algorithm	Hyper-parameter	Searching space	Best parameters
Decision tree^a^	criterion	[gini, entropy]	gini
	max_depth	[2, 4, 6, 8, 10]	8
	min_samples_split	[2, 10, 20, 30, 40]	2
	min_samples_leaf	[1, 10, 20]	20
AdaBoost^b^	learning_rate	[0.001, 0.01, 0.1, 0.5]	0.5
	n_estimators	[10, 50, 100, 200, 300]	300
XGBoost^c^	learning_rate	[0.001, 0.01, 0.1, 0.5]	0.1
	n_estimators	[10, 50, 100, 200, 300]	200
	max_depth	[2, 4, 6, 8, 10]	4
Random forest^d^	criterion	[gini, entropy]	Entropy
	n_estimators	[10, 50, 100, 200, 300]	300
	max_depth	[2, 4, 6, 8, 10]	10

^a^Other default settings: splitter = best; max_features = None; max_leaf_nodes = None.

^b^Other default settings: algorithm = SAMME.R;

^c^Other default settings: min_child_weight = 1; max_delta_step = 0; gamma = 0; colsample_bytree = 1; colsample_bylevel = 1; colsample_bynode = 1; booster = gbtree;

^d^Other default settings: min_samples_split = 2; min_samples_leaf = 1; max_features = auto; max_leaf_nodes = None.

### Evaluation of predictive model performance

To evaluate the performance of each model in predicting unplanned CVD readmission within 180 days, we calculated four performance evaluation measures (i.e., accuracy, precision, recall, F2 score) in the testing set. The F2 score was calculated using the formula: F2 = (1 + 2^2^) × precision × recall/(2^2^ × precision + recall)^24^. We used F2 score because recall is more important than precision; for example, missing a false negative patient is more costly in terms of poor prognosis and healthcare expenditure than reviewing a patient classified as false positive^24^. We also calculated the area under the receiver operator characteristic curve (ROC–AUC) and the area under the precision recall curve (PR–AUC). The precision recall curve is commonly used to evaluate the model performance, especially in studies using imbalanced data^20^. Using a 5 by 2-fold cross-validation approach, we calculated the performance evaluation measures for the models and conducted a paired Student’s t-test^25^. The paired Student’s t-test with Bonferroni correction for multiple comparisons was used to test whether the difference in the mean performance of the models was statistically significant^25,26^. As the hyperparameters were tuned to maximize the AUC, we conducted statistical tests to compare the AUC values between the models. We report the mean and 95% confidence interval (CI) of the performance evaluation measures.

### Evaluation of feature importance

We used the SHapley Additive exPlanations (SHAP) approach to rank the importance of features in the model that demonstrated the highest AUC performance^27^. The SHAP value represents the extent to which a feature contributes to the prediction outcome^28^. We generated the SHAP feature importance plot, summary plot, and cohort bar plot to visualize the results. The summary plot combined feature importance and feature effects, while the cohort bar plot separated the contribution of each feature between different groups, such as men and women.

## Results

### Patient characteristics

We identified a total of 358,629 patients hospitalized for cancer between 2017 and 2018 in the US (mean [SD] age, 62.2 [13.4] years; 184,657 female patients [51.5%]), of whom 21,021 (5.9%) were found to have 180-day unplanned CVD readmission. Sample characteristics and information on all features at the time of index hospitalization for cancer were shown in Table 1.

### Predictive model performance

**Figure 1:**
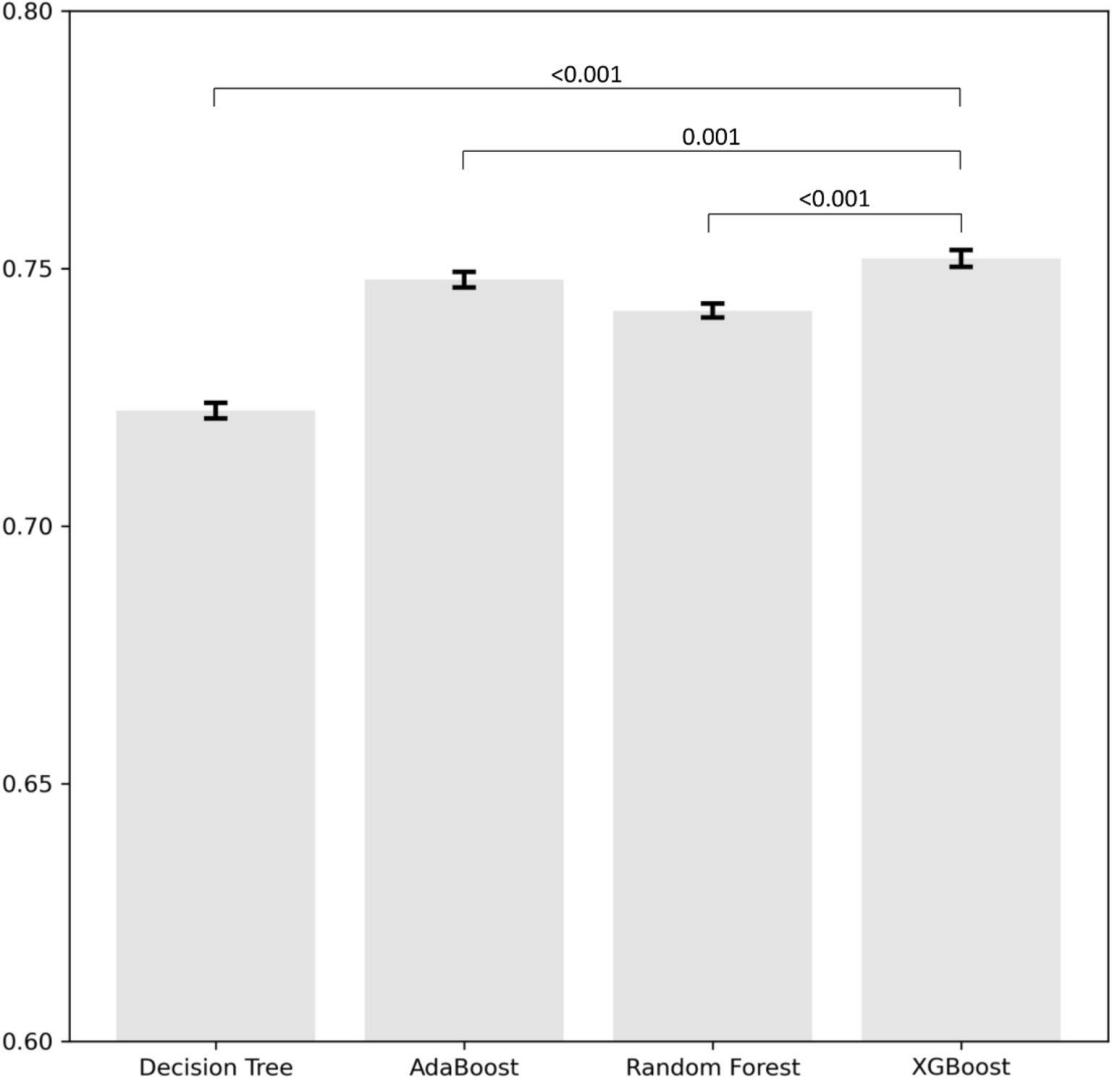
Average area under the curve of the different models using 5 by 2-fold cross-validation. Error bars represent 95% confidence intervals across folds. Comparisons were conducted between XGBoost and three different models.

Figure 1 depicts the bar plot showing the mean AUC and 95% confidence intervals obtained from the 5 by 2-fold cross-validation for the various models. The results indicate that the AUC of XGBoost was significantly higher compared to the other methods. The performance of each algorithm in predicting unplanned readmission due to any CVD among patients hospitalized for cancer was shown in Table 3. Compared to other ML algorithms, the XGBoost approach achieved the highest AUC in both the ROC curve (0.75) and PR curve (0.15), followed by the AdaBoost (ROC–AUC, 0.75; PR–AUC, 0.15) and random forest (ROC–AUC, 0.74; PR–AUC, 0.14), while the decision tree had the lowest AUC (ROC–AUC, 0.72; PR–AUC, 0.13) (Table 3).

**Table 3:** Results of the evaluated machine learning models.

Model	AUC	PR–AUC	Accuracy	Precision	Recall	F2 score
DT	0.722 (0.721–0.724)	0.125 (0.124–0.126)	0.539 (0.529–0.550)	0.095 (0.094–0.096)	0.807 (0.796–0.817)	0.323 (0.322–0.325)
AdaBoost	0.748 (0.746–0.749)	0.147 (0.146–0.149)	0.605 (0.604–0.607)	0.107 (0.106–0.107)	0.776 (0.773–0.780)	0.344 (0.343–0.345)
XGBoost	0.752 (0.750–0.754)	0.152 (0.150–0.154)	0.598 (0.596–0.601)	0.106 (0.106–0.106)	0.787 (0.783–0.790)	0.344 (0.343–0.345)
RF	0.742 (0.740–0.743)	0.140 (0.139–0.142)	0.604 (0.601–0.607)	0.105 (0.105–0.106)	0.769 (0.765–0.774)	0.341 (0.339–0.342)

*AUC* area under the curve, *DT* decision tree, *PR–AUC* area under the precision recall curve, *RF* random forest.

### Feature importance

**Figure 2:**
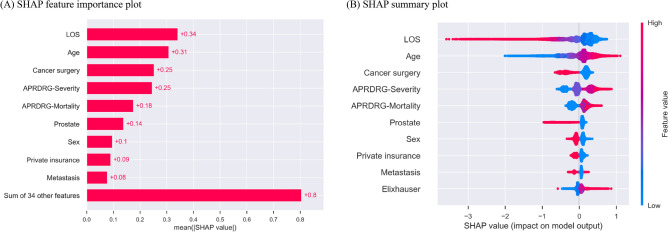
SHAP feature importance and summary plots of the top 10 features from the XGBoost model. (**A**) SHAP feature importance plot. (**B**) SHAP summary plot. The features are ordered according to their importance. Length of stay (LOS) was the most important feature. The SHAP value of a feature represents the extent to which this feature contributes to the prediction result. A positive SHAP value for a feature indicates an increased predicted risk of readmission, while a negative SHAP value indicates a decreased predicted risk of readmission. *SHAP* SHapley Additive exPlanations.

**Figure 3:**
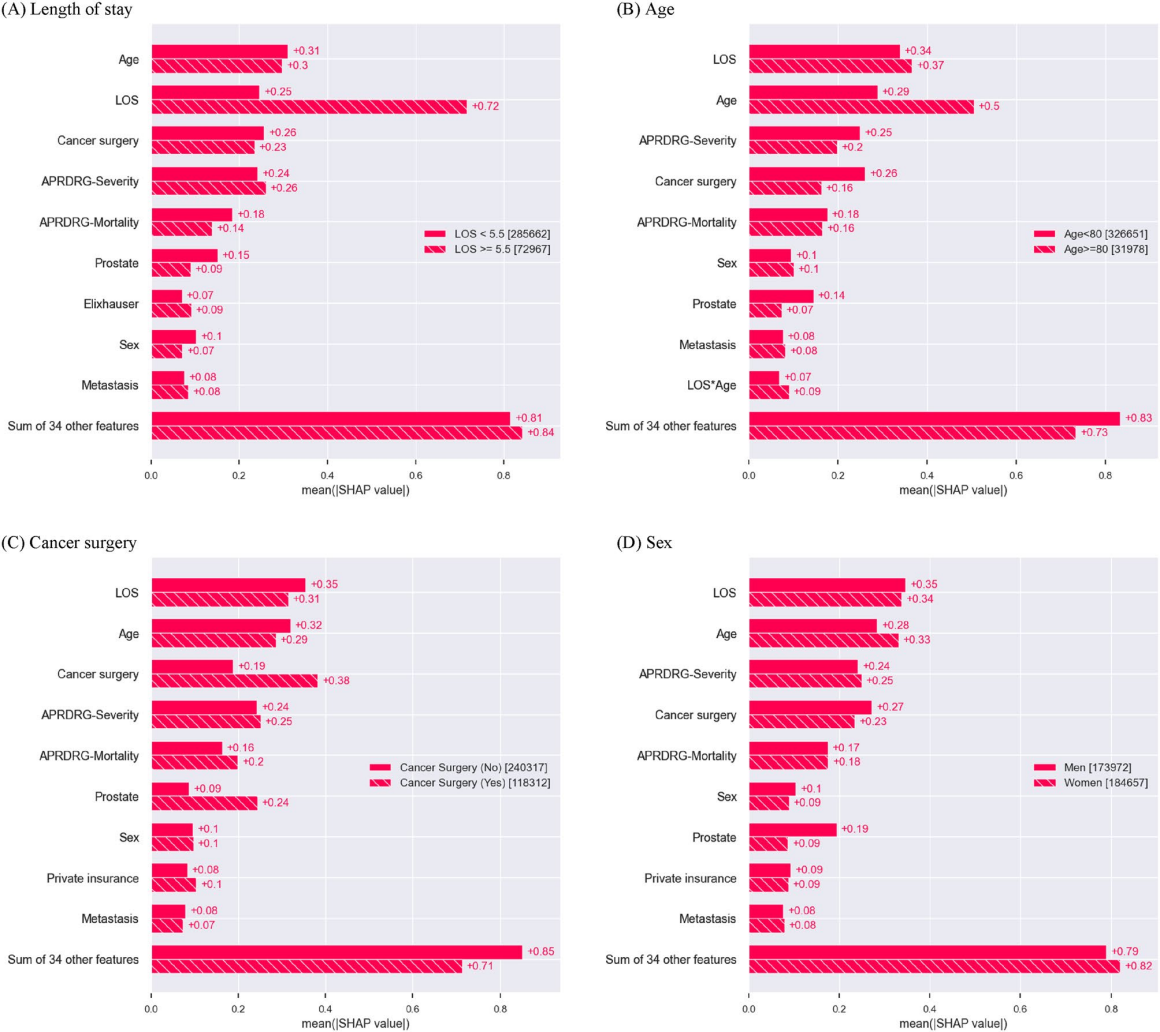
SHAP feature importance by different cohorts. (**A**) Length of stay. (**B**) Age. (**C**) Cancer surgery. (**D**) Sex. SHAP, SHapley Additive exPlanations.

In Fig. 2, we present the SHAP feature importance and summary plot of the top ten features from the XGBoost model, which demonstrate its superior performance in terms of AUC. Among the top ten predictors based on the feature importance, length of stay was identified as the most important predictor, followed by age and cancer surgery. A shorter length of stay, higher age, and no cancer surgery during the index hospitalization contribute to a positive prediction of readmission (readmission occurred). Full results for Fig. 2 are given in Supplementary Figs. 2 and 3. In Fig. 3, we present the SHAP cohort bar plots for the subgroups of length of stay, age, cancer surgery, and sex, which are included in the top ten features from the XGBoost model. Some features showed higher importance for specific subgroups in predicting readmissions. For example, the length of stay feature was more important in the higher age group, the group without cancer surgery during the index hospitalization, and men in predicting readmission.

## Discussions

This population-based study applied ML to predict unplanned readmission for CVD after initial hospitalization for cancer and demonstrated that XGBoost showed a comparatively better predictive performance among four commonly used ML algorithms in the CVD research field. Similarly, other studies compared different ML models in predicting unplanned readmissions using electronic health records or registry data, and XGBoost outperformed the other ML models^29–31^. Our results add to the literature suggesting that XGBoost could be a promising model for predicting the risks of readmissions regardless of the data type.

ML has been tested on several disease states. A few studies used ML to predict various outcomes, including 30-day or 90-day readmissions due to heart failure^29,30,32^. The studies suggested application of ML in predicting risks for CVD-related readmissions is feasible. Previous studies on cancer made use of ML to predict various health outcomes of cancer. A study utilized ML to identify women who were at high risk of developing cervical cancer from screening data collected at a local hospital^33^. Despite the small sample size, ML accurately identified women who have a risk of developing cervical cancer^33^. Another study applied ML to predict patients at risk of short-term mortality^34^. Although the results showed a high risk of bias, ML showed promising performance in predicting short-term mortality in patients^34^. A mini review has identified past studies used of the application of ML to predict the outcomes of cancer such as susceptibility, recurrence, and survival^35^. To our knowledge, this study is the first to use ML methods to explore the CVD-related readmissions for patients hospitalized with cancer in the United States. In our study, the best predictive model was based on XGBoost for the readmission. This finding suggests that applying this model to cancer hospitalizations may help identify patients who need follow-up care to prevent readmission.

Previous studies utilizing ML reported similar risk factors to ours for unplanned readmission in cancer patients. An NRD study analyzed patients who underwent spinal surgery for a metastatic spinal column tumor, and deep vein thrombosis (CVD) was one of the top features of importance for unplanned 30-day readmission^36^. The all-cause unplanned readmission in the study was similar to our feature importances, such as age and kidney disorders^36^. Another NRD study identified risk factors for early readmission after esophagectomy for curative treatment of early-stage esophageal cancer^37^. Similar to our findings, Bolourani and colleagues reported that factors such as age and length of stay were the reasons for all-cause 30-day readmission in cases containing atrial fibrillation and coronary artery disease^37^. A study on hospital-wide all-cause readmission analyzed the electronic health records of 96,550 patients from 205 sites, which provided robust clinical features^26^. The robustness of features resulted in identifying novel risk factors and protective factors for unplanned readmissions^31^. In previous studies, it has been reported that cancer patients have a higher overall risk of coronary heart disease and stroke compared to patients without cancer during the first 6 months through 10 years after cancer diagnosis^12,13^. Our preliminary findings from the HCUP-NRD database have revealed a significantly higher rate of 180-day readmissions among cancer patients compared to non-cancer patients. These findings are currently undergoing revision for submission to Reviews in Cardiovascular Medicine. As a result, we have developed a prediction model for CVD specifically for cancer patients to identify crucial predictors that are relevant to this specific population. Previous evidence suggests that cancer patients may require alternative CVD risk prediction models^38^. Considering that cancer patients are at a higher risk of developing CVD and associated predictors, these findings offer valuable insights into understanding the crucial factors for predicting CVD readmission in this patient population. Our findings regarding risk factors can guide further research aimed to reduce unplanned readmission rates for cancer patients.

We acknowledge several potential limitations in this study. First, some factors that can be strong predictors of hospital readmission are not available in the NRD, such as cancer stages, chemotherapy regimen information, race/ethnicity, or laboratory test results; thus, the impact of these factors could not be assessed. Future effort can be focused on including more predictors to improve the performance of prediction models. Furthermore, although we employed a knowledge/expert-based approach for feature engineering in this study, future research could benefit from adopting a data/model-driven approach. For instance, utilizing Word2Vec to generate feature vectors from ICD codes could provide a wealth of information to further enhance the analysis^39,40^. Second, although we tried to solve the data imbalance, the advanced performance may be limited by the small number of CVD readmission events. To improve prediction performance, future research can continue this effort to test and refine the prediction models by trying different sampling methods, different feature sets, or utilizing additional ML algorithms. Third, although the HCUP routinely performs quality control to confirm the validity and consistency of databases^41^, a possibility of miscoding for cause of readmission may influence the performance of our prediction models. Fourth, we focused only on the 180-day readmission outcome. We developed a prediction model for the occurrence of readmission within 180 days, considering previous evidence indicating that CVD requires a longer follow-up period and that a 180-day timeframe may hold significance^12,13,42,43^. Additionally, we conducted preliminary analyses, which are currently undergoing revision for submission to the Reviews in Cardiovascular Medicine. These initial findings revealed a significantly higher rate of 180-day CVD readmission among cancer patients compared to non-cancer patients. Although survival analyses have not yet been conducted, we plan to explore this aspect in future research endeavors. Fifth, due to the limited number of occurrences for each individual CVD outcome, we were unable to develop separate prediction models for each CVD outcome. Nevertheless, with the use of population-based data, this study provides insights that may help to guide clinical decisions related to preventive strategies for unplanned CVD readmissions among hospitalized cancer patients.

## Conclusions

In conclusion, our study demonstrated the feasibility and performance of the ML approach in predicting the risk of unplanned readmission for CVD after the index hospitalization for cancer. The results suggest that ML, especially XGBoost, can be used as a prognostic prediction model for cancer patients in unplanned care settings in the future. In terms of value-driven healthcare decision making, this study contributes to a better knowledge of the risk prediction of CVD-related unplanned readmissions, which may be used as a basis for facilitating future research on this topic.

### Supplementary Information

Supplementary Figures.

## Data Availability

The data that support the findings of this study are available for purchase from the Central Distributor of the Healthcare Cost and Utilization Project (HCUP). To access the data, other researchers can contact HCUP through the HCUP Central Distributer (https://www.distributor.hcup-us.ahrq.gov) and purchasing the relevant years of HCUP data.
