# Supplementary material for: Predicting unplanned readmission due to cardiovascular disease in hospitalized patients with cancer: a machine learning approach
Source: Sci Rep. 2023 Aug 18;13:13491. doi: 10.1038/s41598-023-40552-4 (PMC10439193; doi:10.1038/s41598-023-40552-4)
Supplement: Supplementary file 1 — Supplementary Figures. [file 41598_2023_40552_MOESM1_ESM.docx]

| 2017-2018 NRD admission for cancer  N=1,022,879  Analytic cohort  N=358,629  Exclusions:   1. Age <18 years old: N=9,476 2. CVD at the index hospitalization: N=291,414 3. Missing values: N=15,964 4. July to December discharge: N=491,011 5. Died during index hospitalization: N=43,294 |
| --- |

**Supplementary Fig 1. Patient selection flow**


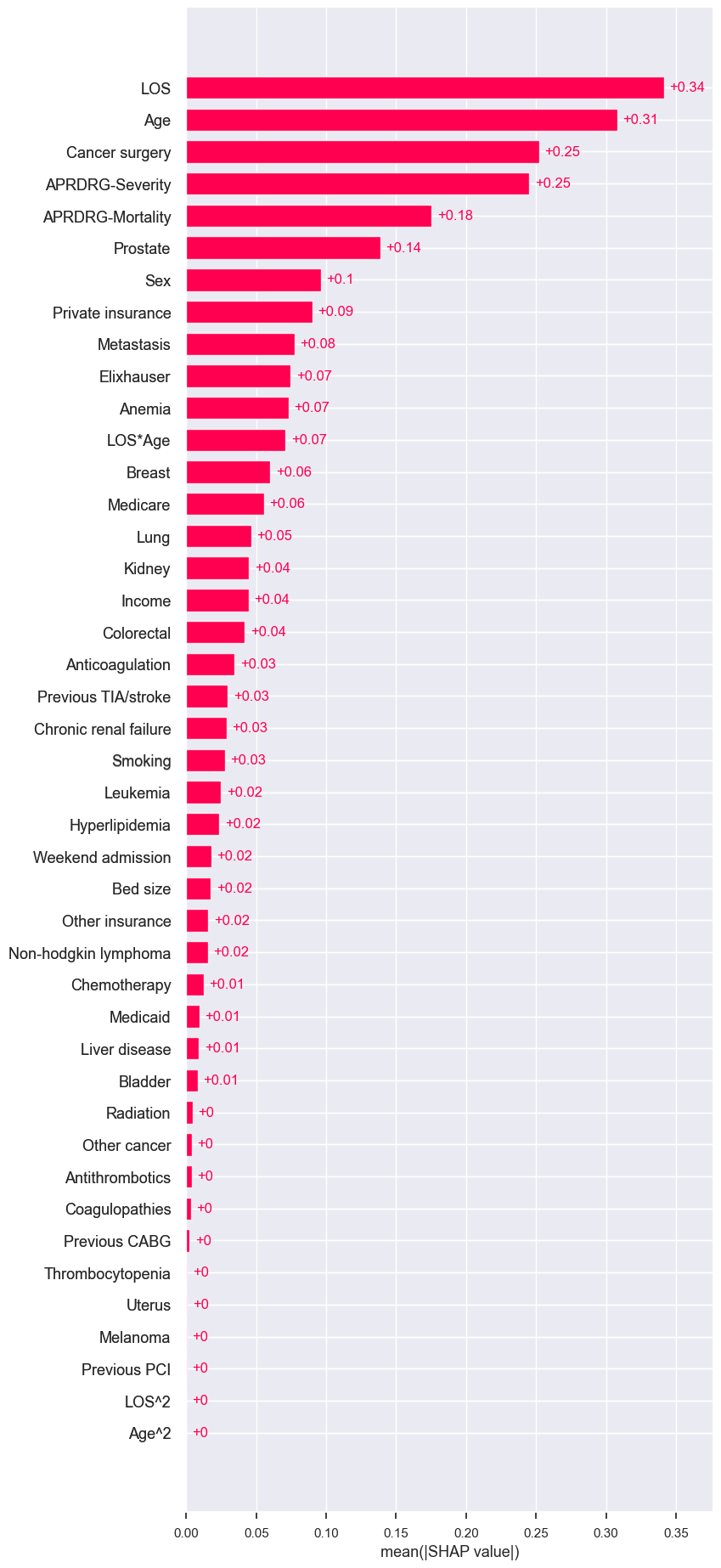


**Supplementary Fig 2. Feature importance of XGBoost ranked by SHAP value.**


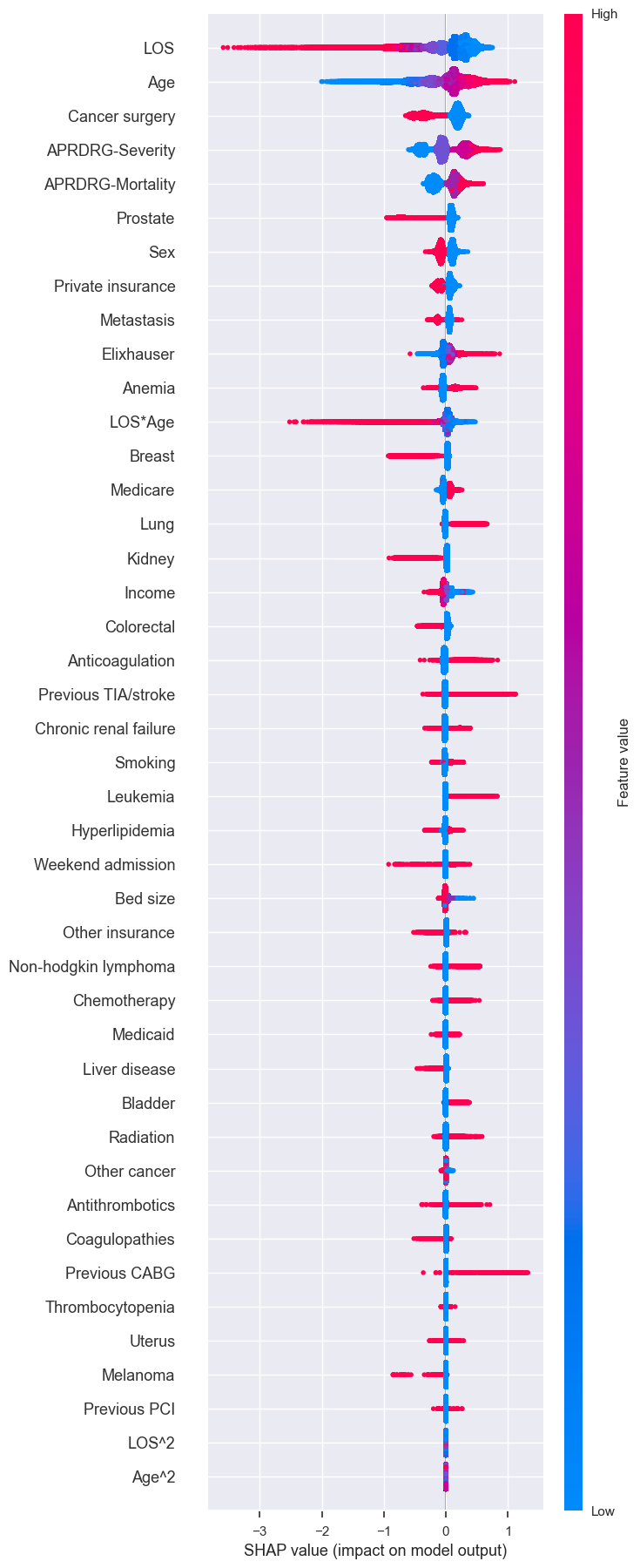


**Supplementary Fig S3. The summary plot utilizes SHAP values from XGBoost.**
